# The impact of pre-existing thyroid diseases on susceptibility to respiratory infections or self-reported sickness during the SARS-CoV-2 pandemic

**DOI:** 10.20945/2359-3997000000436

**Published:** 2022-06-02

**Authors:** Maartje A. J. Rops, Simone J. C. F. M. Moorlag, Rosanne C. van Deuren, Martin Jaeger, Leo A. B. Joosten, Marco Medici, Mihai G. Netea, Jan W. A. Smit, Romana T. Netea-Maier

**Affiliations:** 1 Radboud University Medical Center Division of Endocrinology Department of Internal Medicine Nijmegen Netherlands Department of Internal Medicine, Division of Endocrinology, Radboud University Medical Center, Nijmegen, The Netherlands; 2 Radboud University Medical Center Department of Internal Medicine and Radboud Center for Infectious Diseases Nijmegen Netherlands Department of Internal Medicine and Radboud Center for Infectious Diseases, Radboud University Medical Center, Nijmegen, The Netherlands; 3 Radboud University Medical Center Department of Human Genetics Nijmegen Netherlands Department of Human Genetics, Radboud University Medical Center, Nijmegen, The Netherlands; 4 Erasmus University Medical Center Department of Internal Medicine and Academic Center for Thyroid Diseases Rotterdam Netherlands Department of Internal Medicine and Academic Center for Thyroid Diseases, Erasmus University Medical Center, Rotterdam, The Netherlands; 5 University of Bonn Life & Medical Sciences Institute Department of Immunology and Metabolism Bonn Germany Department of Immunology and Metabolism, Life & Medical Sciences Institute, University of Bonn, Bonn, Germany

**Keywords:** Thyroid dysfunction, COVID-19, SARS-CoV-2

## Abstract

**Introduction:**

This study aimed to evaluate the incidence, severity and presence of symptoms of respiratory tract infections and COVID-19, in patients with pre-existing thyroid dysfunction compared to individuals without thyroid diseases, during the peak month of the COVID-19 pandemic in the Netherlands.

**Subjects and methods:**

In this retrospective observational cohort study, all patients currently under follow-up at the Radboud UMC for thyroid dysfunction received a digital questionnaire. Primary outcomes were incidence of self-reported sickness and cases diagnosed with COVID-19. We compared these primary outcomes between these patients and individuals without thyroid diseases that received the same questionnaire, recruited from the Human Functional Genomics Cohort at the Radboud UMC.

**Results:**

In total, 238 patients with pre-existing thyroid dysfunction and 161 controls were included. Patients did not report more sickness (30.7% vs. 29.2%; p = 0.752) or microbiologically confirmed SARS-CoV-2 infections (1.7% vs. 0.6%; p = 0.351). COVID-19 clinical diagnosis was more frequently made in patients with thyroid diseases (4.2% vs. 0.6%; p = 0.032), despite overall lower incidence of self-reported respiratory related symptoms (52.8% vs. 63.8%; p = 0.028), compared to controls. Sub-group analysis between patients with autoimmune and not-autoimmune thyroid dysfunction did not reveal significant associations with respect to any of the outcome measures.

**Conclusion:**

This retrospective survey of a cohort of patients with from a tertiary academic hospital suggests that pre-existing thyroid dysfunction, independent from the aetiology, does not lead to an apparent risk to develop respiratory tract infections and COVID-19 related symptoms.

## INTRODUCTION

COVID-19 is caused by the ‘severe acute respiratory syndrome coronavirus 2’ (SARS-CoV-2) (1), and although it is a syndrome with a broad spectrum of clinical manifestations, most patients experience respiratory symptoms (2,3). Zheng and cols.'s (4) systematic review showed that males, aged over 65 years, who were smokers had an increased risk of developing critical COVID-19 disease. Furthermore, hypertension, cardiovascular diseases, diabetes and respiratory system diseases were found to be more prevalent in patients with severe COVID-19, compared to patients with mild disease (4,5). This suggests that these comorbidities are important risk factors for more severe disease of COVID-19. Nonetheless, little is known about the risk profile of patients with other comorbidities, such as with thyroid diseases.

Immune responses required for adequate defence against infections are tightly regulated by numerous signals from the nervous and endocrine systems, including thyroid hormones (6–9). Moreover, the immune and the neuroendocrine systems interact by sharing feedback mechanisms and receptors for hormones and cytokines (6,10,11). As such, overt hyperthyroidism is associated with altered antibody production, increased reactive oxygen species production, cell migration, lymphocyte proliferation, and decreased production of pro-inflammatory cytokines (7,8). In contrast, hypothyroidism is associated with altered antibody production, decreased cell migration and lymphocyte proliferation, and potentially an increased susceptibility to infection (8,9,12).

Thyroid diseases are some of the most prevalent endocrine diseases (13). The main mechanism that causes thyroid dysfunction, both hyperthyroidism and hypothyroidism, is autoimmunity against thyroid antigens, which affects more than 5% of the population (14). In these cases, in addition to the direct effects of the thyroid hormone levels, the intricate interactions between different compartments of the immune system leading to the autoimmune status of these patients, or the medication they use (e.g. antithyroid drugs), may also be associated with an impaired immune response.

The present study aims to evaluate the incidence, severity and presence of symptoms of respiratory tract infections, with a specific focus on SARS-CoV-2, in a cohort of patients with pre-existing thyroid diseases causing thyroid dysfunction, during the first peak of the COVID-19 pandemic in the Netherlands. Questionnaire data from the thyroid cohort was compared to data from individuals without thyroid conditions who received the same questionnaire to assess the possible different risk factors that contribute to respiratory tract infections.

## SUBJECTS AND METHODS

### Study population

All patients currently under follow-up at the Radboud University Medical Centre (Radboud UMC) for a thyroid disease causing thyroid dysfunction were approached (n = 369). Exclusion criteria included: insufficient understanding of Dutch language and altered health status that did not allow them to participate. In the patient group, 10 patients reported using the following potentially immunosuppressive medication: prednisolone (two patients), methotrexate (two patients), adalimumab (one patient), etanercept (one patient), vedolizumab (one patient), tocilizumab (one patient), certolizumab (one patient), and imatinib (one patient).

The control cohort consisted of 460 apparently healthy individuals from the same geographic region (Nijmegen) without known thyroid diseases who were recruited from one of the Human Functional Genomics Cohorts at the Department of Internal Medicine of the Radboud UMC (500FG cohort) (15). Participants (both patients and controls) who had previously received a vaccination with BCG (Bacillus Calmette-Guérin) were excluded because of potential interference of this vaccination with the risk of respiratory tract infections and/or infection with SARS-CoV-2 or the severity of the disease (16–18).

This study was approved by the Research Ethics committee at the Radboud UMC Nijmegen, the Netherlands and was performed in accordance with the principles expressed in the Declaration of Helsinki. All participants gave written informed consent for inclusion in the study.

### Study protocol

Primary outcome measures were self-reported sickness, defined as “any illness that led to absenteeism from work or prevented the individual from performing activities outside the house” (19), and confirmed or clinical diagnosis of COVID-19 by a physician. Secondary outcomes were incidence of various COVID-19-related symptoms, and self-reported hospital and intensive care unit (ICU) admissions.

Data was obtained using a digital questionnaire developed in Castor EDC (www.castoredc.com). Questionnaire data from the control cohort had been gathered previously for the purpose of another study (18), and contained information on self-reported sickness and symptoms in the period between February 27, 2020 and April 30, 2020 (16). Patients received the questionnaire by email (n = 344) or by post (n = 25) if no email address was known, and covered the period from January 2020 until June 2020, which included the peak with a two-month window before and after the pandemic's first wave in the Netherlands. Individuals in both cohorts were queried on the self-reported COVID-19-related symptoms and severity, COVID-19 infection status, hospital and ICU admissions in the respective period, demographics and travel status. For the patients, current thyroid function status and specific underlying thyroid disease, other comorbidities and the use of medication were extracted from medical records. Patients with thyroid dysfunction were classified as autoimmune (antibody positive hypo- or hyperthyroidism) or non-autoimmune (e.g. toxic multinodular goitre, solitary toxic adenoma, thyroid carcinoma, iatrogenic hypothyroidism or congenital hypothyroidism) thyroid disorder. Current thyroid function status was defined based on thyroid stimulating hormone (TSH) and free thyroxin (fT4) and/or triiodothyronine (T3) levels according to the institutional reference range.

### Statistical analysis

Questionnaire data was extracted from Castor EDC and analysed using IBM statistics SPSS 25. Additional data from patient records was imported manually. Differences between characteristics within the patients’ cohort and various variables between the two study cohorts were assessed using Chi-square or Fisher's exact tests in the case of nominal/categorical data and independent t-tests, or when appropriate, non-parametric equivalent Wilcoxon-rank sum test, for continuous data.

To determine whether the patients were differentially affected by the COVID-19-related disease than the controls, we created a logistic regression model using self-reported sickness as the independent variable and autoimmune thyroid disease (yes/no) as main predictor, next to the possible confounders of age, presence of comorbidities (none/one or more), medication use (none/one or more), healthcare-work (yes/no), known contact with COVID-19 patients (yes/no), and international travel during the study period (yes/no). As we observed a difference in the duration of sickness between the two cohorts, we next aimed to assess what predictors would underlie this difference, such as age, autoimmune thyroid disease (yes/no), current thyroid function status, presence of comorbidities (none/one or more), use of medication (none/one or more) and BMI, which are known potential risk modifiers. Data are shown as mean (±SD) and absolute numbers (percentage). The level of significance was set as a two-tailed p < 0.05.

## RESULTS

Of the 369 patients with pre-existing thyroid dysfunction approached for this study, 238 (64.5%) completed the questionnaire. In the control group, of the 460 volunteers who were sent the questionnaire, 207 (45%) responded. Of these, 44 were excluded because they had been vaccinated with BCG, and 2 because they used levothyroxine. Finally, 161 individuals were included in the control group. The study populations’ characteristics are shown in Table 1. Patients were older (51.0 *vs.* 35.6 years; p < 0.001), more frequently female (75.2% *vs.* 60.9%; p = 0.002), and more of them had comorbidities (p < 0.001) or used medication (p < 0.001) compared to the controls. They also reported less international traveling during the study period (p = 0.001), less frequently worked in the healthcare industry (p < 0.001) and were more frequently unemployed (p < 0.001) compared to the controls. Of the patients, 71 (29.8%) had an autoimmune thyroid disease. At the time of assessment, only 14.3% had clinically manifest hypo- or hyperthyroidism (Table 1). Of the patients with non-autoimmune thyroid diseases, 115 had a history of thyroid carcinoma (aged 54.3 years ± 14.6, 68.7% females) which included 76.5% papillary, 9.6% follicular, 13.9% medullary and the remaining other histological subtypes. Of these, 84.3% were in remission or only had biochemical evidence of disease, 12.2% had structural disease and in 3.5%, the disease status was indeterminate.

**Table 1:** Baseline patient characteristics

Characteristics			Total	Pre-existent thyroid diseases	Controls	p-value
			(n = 399)	(n = 238)	(n = 161)	
Sex						0.002
	Male		122 (30.6)	59 (24.8)	63 (39.1))	
	Female		277 (69.4)	179 (75.2)	98 (60.9)	
Age			44.8 (±17.0)	51.0 (±15.3)	35.6 (±15.0)	<0.001
1 or more housemates			343 (86.0)	206 (86.6)	137 (85.1)	0.680
Outside working housemates			178 (44.6)	118 (49.6)	60 (37.3)	0.015
Occupation						
	Unemployed*		119 (29.8)	90 (37.8)	29 (18.0)	<0.001
	Health care		103 (25.8)	43 (18.1)	60 (37.3)	<0.001
Vaccination status						
	Flu vaccine		107 (26.8)	64 (26.9)	43 (26.7)	0.968
	Other		54 (13.5)	27 (11.3)	28 (16.8)	0.120
Comorbidities						
	None		175 (43.9)	69 (29.0)	106 (65.8)	<0.001
	1 or more		224 (56.1)	169 (71.0)	55 (34.2)	
Use of medication						<0.001
	None		130 (32.6)	9 (3.8)	121 (75.2)	
	1 or more		269 (67.4)	229 (96.2)	40 (24.8)	
Currently smoking			30 (7.5)	17 (7.1)	13 (8.1)	0.729
International traveling**			116 (29.1)	53 (22.3)	63 (39.1)	<0.001
Thyroid disease						
	Autoimmune		NA	71 (29.8)	NA	
		Hypothyroidism	NA	26 (36.6)	NA	
		Hyperthyroidism	NA	45 (63.4)	NA	
	Non-autoimmune		NA	167 (70.2)	NA	
		Hypothyroidism	NA	153 (91.6)	NA	
		Hyperthyroidism	NA	14 (8.4)	NA	
Current thyroid function***						
	Euthyroidism		NA	143 (60.1)	NA	
	Hypothyroidism		NA	3 (1.3)	NA	
	Hyperthyroidism		NA	31 (13.0)	NA	
	Subclinical hypothyroidism		NA	13 (5.5)	NA	
	Subclinical hyperthyroidism		NA	48 (20.2)	NA	

Data are given as absolute numbers (percentage) or mean (± standard deviation). X: data unknown. NA: not applicable.

* This also included being a student, retired or long-term absence due to illness.

** In the 20 weeks previous to the questionnaire.

*** Current thyroid function status was classified as euthyroidism (TSH and fT4 within institutional reference range), hypothyroidism (increased TSH with decreased fT4 and/or T3), hyperthyroidism (decreased TSH with increased fT4 and/or T3), subclinical hypothyroidism (increased TSH with fT4 and/or T3 within the institutional reference range) or subclinical hyperthyroidism (decreased TSH with fT4 and/or T3 within the institutional reference range).

**Figure 1:**
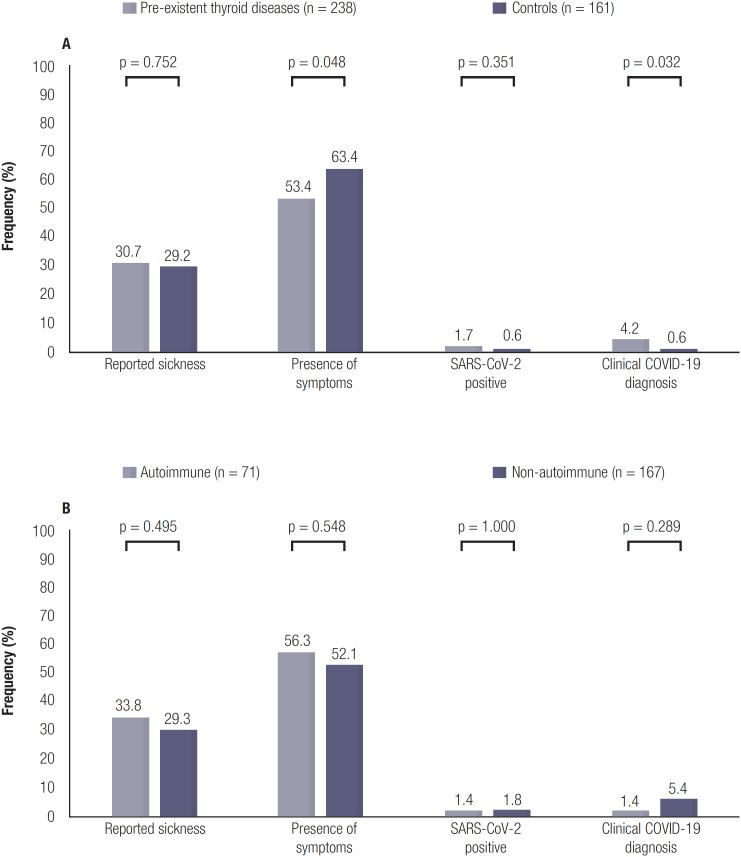
Incidence of reported sickness, COVID-19 related symptoms and confirmed or suspected SARS-CoV-2 infections. P = Chi-square or Fisher's exact p-value. (**A**). Percentage of patients with reported sickness, symptoms of COVID-19, confirmed or suspected SARS-CoV-2 infections in the thyroid dysfunction (n = 238) and control cohort (n = 161). (**B**). Percentage of patients with reported sickness, symptoms of COVID-19, confirmed or suspected SARS-CoV-2 infections in the autoimmune (n = 71) and not-autoimmune cohort (n = 167).

Among the patients, 73 (30.7%) reported sickness for 1 or more days, compared to 47 controls (29.2%, p = 0.752; shown in Figure 1A). No statistically significant difference in number of cases with microbiologically confirmed SARS-CoV-2 was found between the two cohorts (1.7% *vs.* 0.6%, p = 0,351), but the patients were more frequently clinically diagnosed with COVID-19 by a physician (4.2% *vs.* 0.6%, p = 0.032). No hospital or ICU admissions were reported in either of the cohorts.

**Figure 2:**
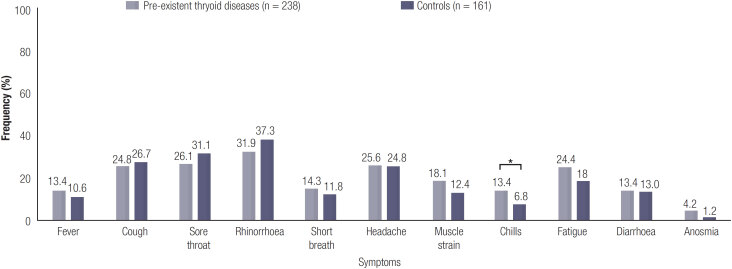
Incidence of various COVID-19 related symptoms. Percentage of patients that reported the various symptoms in the thyroid dysfunction (n = 238) and the control cohort (n = 161). P = Chi-square or Fisher's exact p-value. *p = 0.037.

Controls more frequently reported respiratory tract-related symptoms in general as compared to the patients (63.4% *vs.* 53.4%, p = 0.048), although upon investigating the specific symptoms separately, the patients more frequently reported having chills compared to the controls (13.4% *vs.* 6.8%, p = 0.037) (shown in Figure 2).

Among the patients, no differences were found between those with autoimmune or non-autoimmune disease for any outcome measures (shown in Figure 1B). Furthermore, patients with thyroid carcinoma did not report more sickness (24.3% *vs.* 29.2%, p = 0.372), or more confirmed (0.8% *vs.* 0.6%, p = 1.000) or suspected SARS-CoV-2 infections (4.3% *vs.* 0.6%, p = 0.085) than the control cohort. However, patients with thyroid carcinoma less frequently reported COVID-19-related symptoms compared to individuals in the control cohort (44.3% *vs.* 63.4%, p = 0.002).

The logistic regression model fit created to adjust for possible confounding factors for a correlation between thyroid dysfunction and reported sickness was appropriate (p = 0.616) and showed an AOR of 0.984 (95% CI [0.969–<1.000], p = 0.049) for age.

**Figure 3:**
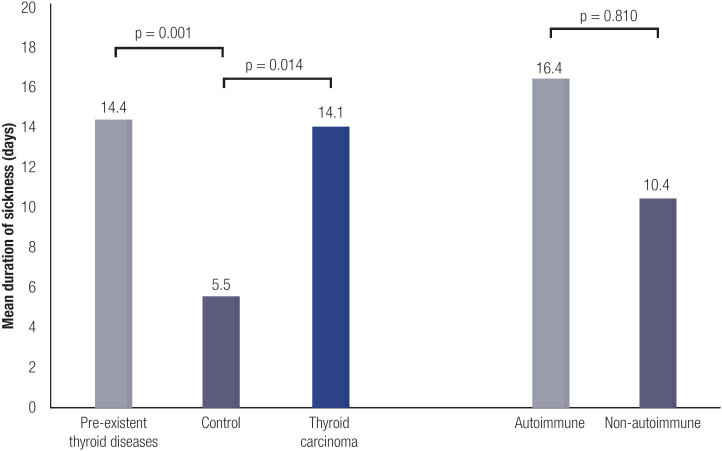
Mean duration of sickness. The average number of reported days of sickness in the total cohort of patients with pre-existing thyroid diseases (n = 238), controls (n = 161), patients with thyroid carcinoma (n = 115), autoimmune (n = 71) and not-autoimmune thyroid diseases (n = 167). P = Wilcoxon rank sum p-value.

The patients reported a longer mean duration of sickness than the controls (14.4 days *vs.* 5.5 days, p = 0.001; shown in Figure 3). Mean duration of sickness was 16.4 days in patients with autoimmune thyroid disease and 10.4 days in patients with non-autoimmune disease (p = 0.810; shown in Figure 3). Patients with thyroid carcinoma also reported a longer duration of sickness (14.1 days) than the controls (5.5 days, p = 0.014). After adjusting in a linear regression model for possible confounders, we could not confirm the significant difference in duration of disease between patients and controls (Table 3).

**Table 2:** Logistic regression model for reported sickness

		95% CI for Exp (B)		
	OR	Lower	Upper	p-value
Constant	0.508	NA	NA	0.082
Age, years	0.984	0.969	<1.000	0.049
Autoimmune thyroid disease	1.094	0.612	1.956	0.761
Healthcare worker	0.849	0.507	1.424	0.536
Medication ≥1	1.721	0.966	3.066	0.065
Comorbidities ≥1	1.119	0.691	1.815	0.647
International travelling*	1.419	0.883	2.282	0.149
Known contact with COVID-19 patient	1.518	0.869	2.653	0.142

Chi-Square 14.189, p-value = 0.048. Lemeshow-Hosmer p-value = 0.616. Nagelkerke R2 = 0.050. OR: odds ratio. NA: not applicable.

* In the 20 weeks previous to the questionnaire.

**Table 3:** Linear regression model for duration of sickness

		95% CI for Exp (B)		
	Beta	Lower	Upper	p-value
Constant	-16.265	-35.122	2.591	0.090
Age, years	0.185	-0.105	0.475	0.209
Autoimmune thyroid disease	-0.954	-10.522	8.614	0.844
Medication ≥1	2.610	-6.883	12.103	0.587
Comorbidities ≥1	4.447	-3.263	12.157	0.256
BMI	0.625	-0.147	1.396	0.111
Hypothyroidism*	--	--	--	--
Hyperthyroidism	1.677	-11.159	14.512	0.796
Subclinical hypothyroidism	-5.804	-23.416	11.807	0.515
Subclinical hyperthyroidism	-5.976	-19.679	7.728	0.390

F-test = 1.430, p-value = 0.188. Adjusted R^2^ = 0.027.

* No patients included in this analysis had clinically manifest hypothyroidism.

## DISCUSSION

We have assessed differences in self-reported sickness or incidence of respiratory or COVID-19-related symptoms, confirmed SARS-CoV-2 infections between patients with pre-existing thyroid dysfunction and controls, or between patients with different aetiologies of thyroid dysfunction. There was no difference in self-reported sickness between patients and controls. Microbiologically confirmed COVID-19 did not differ between the two groups, and the percentages of patients with confirmed COVID-19 were low. Nonetheless, patients with pre-existing thyroid dysfunction were more often diagnosed clinically with COVID-19 by a physician. The differences between microbiological and clinical diagnosis of COVID-19 may have resulted from the national policy for diagnosing COVID-19 during the first wave of the pandemic in the Netherlands, during which only hospitalized patients and medical personnel were tested microbiologically for SARS-CoV-2 due to the shortage of diagnostic tests. In addition, regardless of the underlying thyroid disease, patients with pre-existing thyroid dysfunction reported a longer duration of sickness, possibly related to a more advanced age and comorbidity.

Thus far, only few studies have assessed the susceptibility for respiratory tract infections and/or SARS-CoV-2 infections during the recent SARS-CoV-2 pandemic in patients with pre-existing thyroid dysfunction. Brix and cols. (20) reported using population-based data from Denmark that pre-existing hypothyroidism or hyperthyroidism was not associated with an increased risk of SARS-CoV-2 infection or a worse outcome of the disease. Also, Daraei and cols. (21) found that in a small series of 21 patients with hypothyroidism among a cohort of 390 patients admitted in the hospital with COVID-19 there was no significant difference in mortality between patients with and without hypothyroidism. Similarly, van Gerwen and cols. (22) reported a similar outcome in 251 patients with hypothyroidism versus 3,452 patients without hypothyroidism admitted with COVID-19 in terms of hospitalization, need of mechanical ventilation and mortality. The present study suggests that pre-existing thyroid dysfunction may not lead to an apparent risk in developing respiratory tract infections and COVID-19-related symptoms apart from more frequently reported chills. Chills are highly specific for an infection when correctly recognized. However, in our study, the reported chills could not be objectivated. Nonetheless, the percentage patients a physician diagnosed with COVID-19 was higher among the patients than in controls. This might indicate that patients with pre-existing thyroid dysfunction might bare a modified risk for this infection. Interestingly, the patients and controls also displayed proportions of anosmia, a more pathognomonic COVID-19 symptom, which were comparable to the proportions of those clinically diagnosed with COVID-19. This suggests that the clinical diagnosis was correct in most of these patients. However, due to the lack of microbiological confirmation of the infection, we cannot draw a definitive conclusion on this issue.

One remarkable observation is the higher incidence of self-reported symptoms in the control cohort. One possible explanation could be that the symptoms associated with COVID-19 are very often reported in many other respiratory tract infections and even allergies (considering that the follow-up with the patients was during the spring season), and thus the parameter of clinical symptoms is likely to have been non-specific.

The patients reported a prolonged duration of sickness compared to the controls. Unfortunately, our linear regression model adjusting for possible confounders was unable to confirm this difference, although we hypothesize the lack of power could have caused this because the sub-group analysis included only 128 participants who reported sickness.

Several limitations should be considered. First, because of the study's retrospective nature and the fact that it was based on self-reported information, recall bias cannot be ruled out. Second, due to the small size of the subgroups of patients with different thyroid diseases, we cannot exclude how this study lacked the statistical power to analyse the effects of different thyroid dysfunction aetiologies. Third, because of the pandemic's quickly changing dynamics, data for the two cohorts covered a slightly different, though largely overlapping, period. This may have led to differences in the incidence of the self-reported data. Furthermore, although conclusions on the incidence of confirmed SARS-CoV-2 infection are valid, some of the infected patients and controls may not have been tested because they did not fulfil the case definition at that time. Therefore, the true incidence of SARS-CoV-2 infections is likely underestimated. Another limitation regards using potentially immunosuppressive medication in 10 patients, which could have modified the risk for the disease. However, the linear regression analysis did not reveal a significant effect of the use of medication on the study's main outcome. Moreover, the study population does not reflect the epidemiological data of the general population with thyroid dysfunction, rather it reflect the selected population currently under treatment and follow-up at our tertiary academic centre, including patients with more complex endocrine problems.

In conclusion, this is the first study on self-reported respiratory tract and SARS-CoV-2-related complaints and infection rates during the COVID-19 pandemic in patients with pre-existing thyroid dysfunction. Despite its potential limitations inherent to the retrospective nature of this questionnaire study, these data show that pre-existing thyroid dysfunction, regardless of aetiology, does not lead to an apparent risk to develop respiratory tract infections and COVID-19-related symptoms. Nonetheless, a modified susceptibility to SARS-CoV-2 infection cannot be completely ruled out. Future studies including larger sub-groups of patients with different thyroid diseases and more accurate confirmation of the infection should be conducted to provide more insight into the risk factors of COVID-19 in patients with pre-existing thyroid dysfunction.
